# β-Blockers and Erectile Dysfunction in Heart Failure. Between Myth and Reality

**DOI:** 10.31083/j.rcm2305173

**Published:** 2022-05-13

**Authors:** Sara Corradetti, Giovanna Gallo, Michele Correale, Massimo Piepoli, Roberto Badagliacca, Savina Nodari, Piergiuseppe Agostoni, Damiano Magrì

**Affiliations:** ^1^Department of Clinical and Molecular Medicine, Sapienza University of Rome University, 00189 Rome, Italy; ^2^Department of Cardiology, University of Foggia, 71122 Foggia, Italy; ^3^UOC Cardiologia, G da Saliceto Hospital, 29121 Piacenza, Italy; ^4^Dipartimento di Scienze Cardiovascolari, Respiratorie, Nefrologiche, Anestesiologiche e Geriatriche, Sapienza University of Rome, 00161 Rome, Italy; ^5^Division of Cardiology, University of Brescia, 25121 Brescia, Italy; ^6^Department of Clinical sciences and Community health, Cardiovascular Section, University of Milano, 20122 Milano, Italy; ^7^Centro Cardiologico Monzino, IRCCS, 20138 Milano, Italy

**Keywords:** heart failure, erectile dysfunction, beta-blocker, therapy

## Abstract

Erectile dysfunction (ED) is a major concern in heart failure (HF) due its high prevalence as well as its negative impact on the quality of life, this condition being usually unrecognized and thus untreated. A number of possible causes might contribute to the above mentioned tight association, i.e., shared risk factors, comorbidities and several physiologic HF abnormalities such as impaired exercise tolerance, psychogenic factors and neurohumoral, metabolic and vascular changes. Medications have been blamed for playing also a pivotal role in the ED occurrence and, particularly, the β-blockers. Remarkably, the underlying mechanisms have not been fully identified. All the available scientific literature dealing with this topic derives from studies not addressing this issue in HF, but in other settings, (e.g., arterial hypertension) and are also characterized by important methodological flaws. Thus, given the solid evidences arguing in favor of β-blockers in HF in terms of morbidity, mortality and quality of life, β-blockers at the maximal tolerated dosage in this patients’ category should be recommended, regardless of ED. However, the ED-related issues should not be neglected, and adequate psychological counseling and management should be provided, pursuing the correction of risk factors, the choice of more suitable medications and, in selected cases, adopting specific drugs or devices. The purpose of this narrative review is to highlight the close relationship between ED and HF and, specifically, to focus on a possible β-blockers’ role in determining or, at least, worsening this condition.

## 1. Erectile Dysfunction: Definition and Underlying Mechanisms

The erectile dysfunction (ED) is defined as the consistent or recurrent inability to achieve and/or maintain an erection sufficient to permit satisfactory sexual performance [1]. The ED can be classified based on etiology as psychogenic, organic or mixed psychogenic and organic, the latter being the most common one. The organic form may be associated with neurological disorders, androgen deficiency, vascular causes such as penile arterial insufficiency or veno-occlusive dysfunction, obesity, diabetes mellitus or other systemic disease and, finally, drugs, cigarette smoking or chronic alcoholism. In addition, sexual function progressively declines with aging [2, 3]. Any alteration in the pathway briefly described below might lead to ED (Table 1).

**Table 1.** **Common causes and underlying mechanisms of erectile dysfunction**.

Cause		Mechanism
Psychogenic	Depression	Decreased libido
	Anxiety	Increased sympathetic tone
	Psychological stress	Impaired NO release
Vasculogenic	Hypertension	Decreased arterial flow
	Diabetes	Endothelial dysfunction
	Atherosclerosis	Increased endothelin 1 or noradrenalin
	Impaired vasomotion	Decreased prostacyclin
Neurogenic	Stroke or Alzherimer’s disease	Failure to initiate nerve impulse
	Spinal cord or pelvic injury	Interrupted neural transmission
	Diabetes	Peripheral neuropathy
Hormonal	Hypogonadism	Loss of libido
	Hyperprolactinemia	Inadequate NO release
Drug-induced	SSRI	Central suppression
	β-blockers	Unknown mechanism
	Digoxin	Smooth muscle sodium-pump inhibition
	Spironolactone	Androgen suppression
	Diuretics	Unknown mechanism
	Cigarette smoking	Vascular insufficiency
	Alcohol abuse	Alcoholic neuropathy

NO, nitric oxide; SSRI, selective serotonin reuptake inhibitors.

**Fig. 1.**
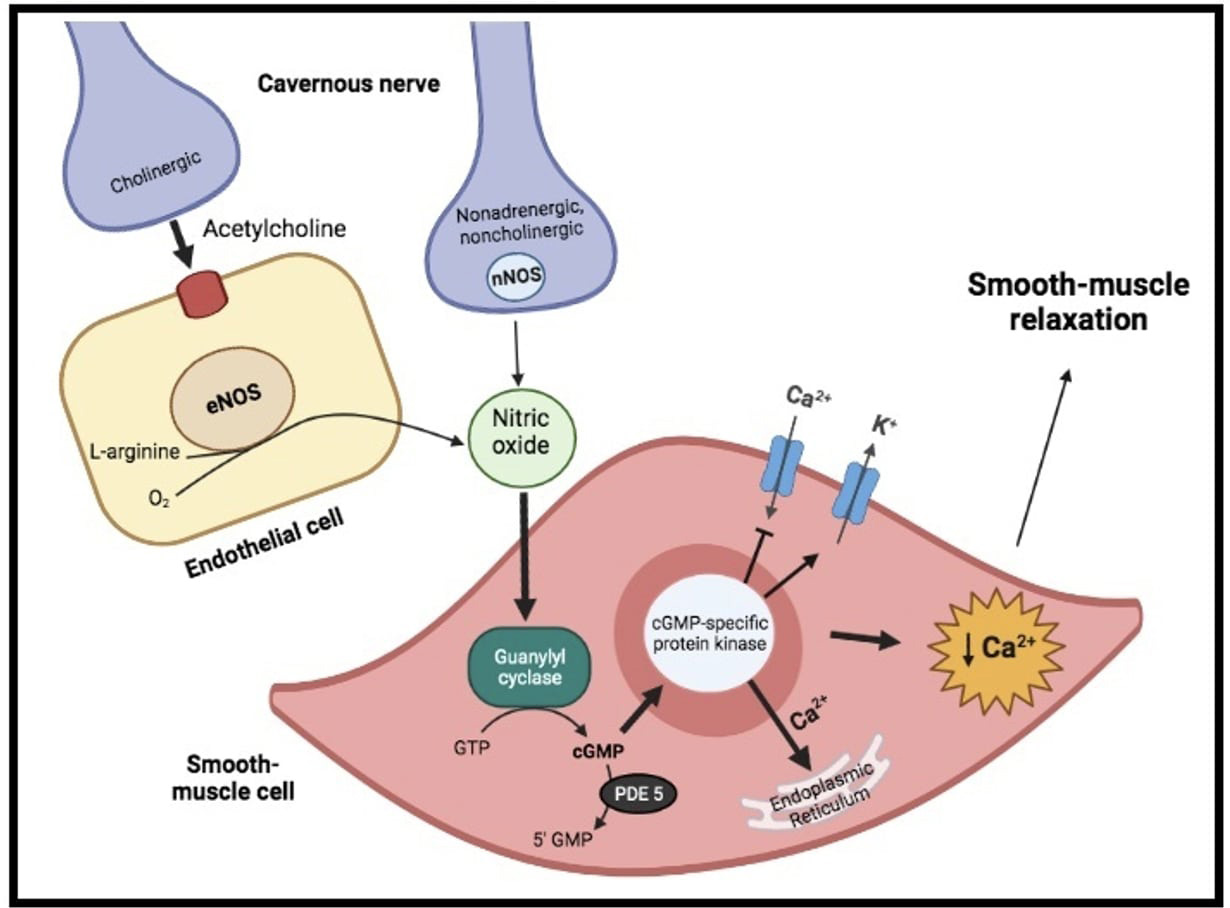
**Physiology of erectile function**. On sexual stimulation, the cavernous nerve terminals release neurotransmitters resulting in production of nitric oxide (NO). Within the smooth muscle cells, it stimulates a soluble guanylyl cyclase, which increases the production of cyclic guanosine monophosphate (cGMP), the intracellular second messenger mediating smooth muscle relaxation. Indeed, cGMP activate a specific protein kinase, leading to opening of potassium ($K^{+}$) channels, closing of calcium channels and sequestration of intracellular calcium (${\mathrm{Ca}}^{2+}$) by the endoplasmic reticulum. The resultant fall in intracellular calcium leads to smooth-muscle relaxation.

Physiologically, penile erection results from the integrative synchronized action of neuronal and vascular systems, both of them being modulated by psychological factors and hormonal status. Indeed, on sexual stimulation, the cavernous nerve terminals release neurotransmitters resulting in relaxation of the trabecular smooth muscle and vasodilation of the arteries and arterioles supplying the erectile tissue (Fig. 1). Thus, penile blood flow extremely increases and sinusoidal spaces rapidly expands. In addition, the enlargement of the sinusoids compresses the subtunical venular plexuses against the tunica albuginea decreasing the venous outflow to a minimum. A cessation of neurotransmitters release, the metabolization of second messengers by phosphodiesterase or sympathetic discharge during ejaculation (i.e., adrenergic receptors’ activation on the cavernous arteries and trabecular smooth muscles) can lead to detumescence. During this phase, contraction of the trabecular smooth muscle allows venous drainage of the lacunar spaces and relief of the erection [4]. In such a context, nitric oxide (NO), released from parasympathetic nerve terminals and vascular endothelium, is probably the principal neurotransmitter involved. Indeed, within the smooth muscle cells, NO stimulates a soluble guanylyl cyclase, which in turn increases the production of cyclic guanosine monophosphate (cGMP), the intracellular second messenger mediating smooth muscle relaxation. Thereafter, cGMP activates a specific protein kinase leading to phosphorylation of certain proteins to cause opening of potassium channels, closing of calcium channels and sequestration of intracellular calcium by the endoplasmic reticulum. The resultant fall in intracellular calcium leads to smooth muscle relaxation, that is essential for maximal penile engorgement. Eventually, during the return to the flaccid state, cGMP is metabolized by type 5 phosphodiesterase (PDE-5), resulting in detumescence [4, 5].

In this narrative review we sought to analyze the pathophysiological mechanisms involved in the development of ED in patients with heart failure (HF) and, particularly, we tried to answer a historical question on a possible detrimental role of β-blockers on erectile function in this setting.

## 2. Erectile Dysfunction in Heart Failure: Prevalence and Risk Factors

Several studies sought to investigate the overlap between ED and HF, showing a prevalence ranging from 60% to 75%, regardless of the HF etiology [6, 7]. In general, patients suffering from HF experience a decrease in libido and in frequency of coitus, negative changes in sexual performance and a general dissatisfaction related to their sexual function. Even, it has been reported that about one quarter of them cease all sexual activity [8]. However, despite the well-known tight association between these conditions, cardiologists address rarely the presence of an ED concern in contrast with patients’ expectations who would like their physicians to be interested in this issue [9]. Accordingly, a large percentage of HF patients remains without a diagnosis and thus untreated.

**Fig. 2.**
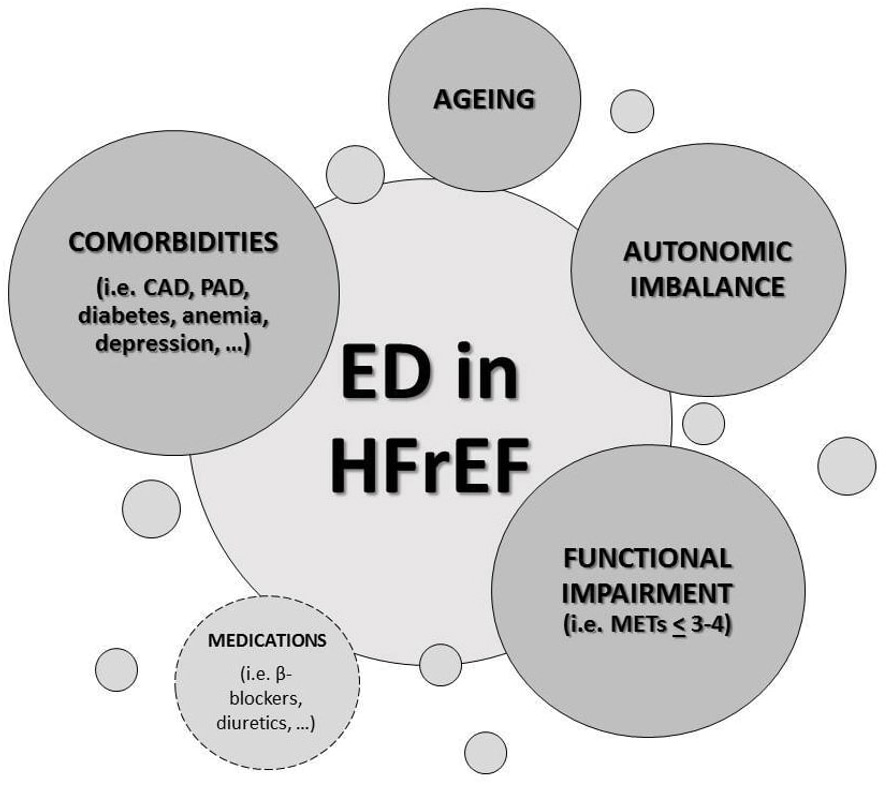
**Causes of erectile dysfunction (ED) in heart failure with reduced ejection fraction (HFrEF)**. ED, erectile dysfunction; HFrEF, heart failure with reduced ejection fraction; CAD, coronary artery disease; PAD, peripheral artery disease; METs, metabolic equivalents of task (1 MET = 3.5 mL/kg/min $O_{2}$ uptake).

A number of possible reasons may contribute to the high ED incidence in HF patients (Fig. 2). Firstly, depression and anxiety, usually described conditions in HF, may play a pivotal role in sexual dysfunction as well as the concomitant treatments with selective serotonin reuptake inhibitors (SSRIs), whose sexual side effects are well known [6], could magnify the issue. Another important contributing cause for ED in this setting is the exercise impairment degree, sexual function being related with New York Heart Association (NYHA) functional class, the 6-minute walk test and the peak oxygen uptake (${\mathrm{pVO}}_{2}$) [8]. Indeed, the physical component of sexual activity (i.e., the orgasmic phase) requires at least 3–4 metabolic equivalents of task (MET), thus it is reasonable an impaired sexual function for all those patients with a ${\mathrm{pVO}}_{2}$ lower than 10–15 mL/min/kg [8, 10]. Third, ED and HF are tightly associated due to the coexistence of shared risk factors and comorbidities, such as ageing, obesity, altered lipid profile, hypertension, anemia, diabetes mellitus and cigarette smoking [11, 12]. Accordingly, atherosclerosis, which accounts for approximately 40% of ED in men over 50 years old, is one of the most common causes of cardiomyopathy in high income countries. Furthermore, HF is associated with a neurohumoral imbalance which undoubtedly contribute to endothelial dysfunction and, consequently, to ED. In such a context, studies evaluating whether or not NO production is altered in HF are conflicting, but it has been demonstrated that there are increased levels of circulating vasoconstrictors such as endothelin, as well as a reduction in vasodilators such as prostacyclin [6]. Finally, many of the medications used in HF therapy have been associated with an erectile function impairment [6].

## 3. β-Blockers and Erectile Dysfunction: Underlying Mechanisms and Scientific Evidence

Historically, β-blockers have been considered as a class of medications detrimental with respect the sexual function. Nonetheless, in spite of many attempts to explain this kind of relationship, the underlying pathophysiological mechanisms have not been clearly identified. Furthermore, it should be remarked that there is lack of studies specifically addressing this matter in the HF setting but only in hypertensive patients. Conversely, the β-blockers’ treatment represents a mainstay of the HF treatment, particularly in those with reduced ejection fraction (HFrEF) where it exerts an undoubted favorable prognostic impact [13]. Indeed, the CIBIS-II (bisoprolol), the COPERNICUS (carvedilol), the MERIT-HF (metoprolol) and the SENIORS (nebivolol) trials all demonstrated that β-blockers therapy is more effective than placebo in HFrEF patient in terms of overall mortality, cardiovascular mortality as well as of number of hospitalizations [14, 15, 16, 17].

**Fig. 3.**
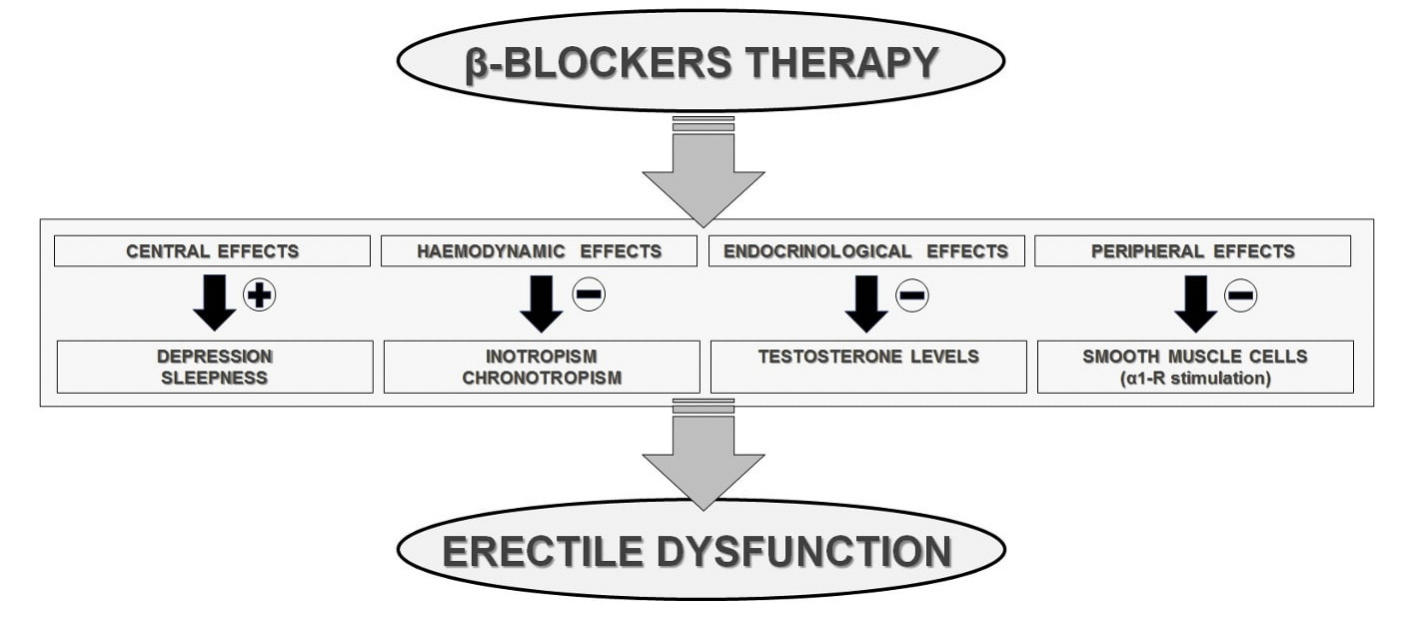
**
β-blockers and erectile dysfunction: mechanisms possibly implied**.

From a pathophysiological viewpoint, due to their block of the receptor sites for the endogenous catecholamines, the β-blockers’ negative effect is primarily thought to be mediated by the inhibition of the sympathetic nervous system, which is involved in the integration of an erection and the stimulation of testosterone release. Similarly, given their inotropic/chronotropic negative effect, they may also cause ED through decreasing perfusion pressure by a drop in blood pressure. Another theory, even if not yet proven [18], calls for both a direct and indirect effects on cavernosal smooth muscle increasing contraction due to unopposed alpha-receptor stimulation. Furthermore, throughout their effect on luteinizing hormone, β-blockers might induce a depression of Leydig cell activity leading a reduction in testosterone levels, which have been demonstrated to be necessary for maintenance of intra-penile NO synthase levels [19]. In addition, β-blockers can adversely affect sexual performance by increasing the latency to initial erection and reducing the number of erectile reflexes [20]. Last, β-blocker therapy may cause sleepiness or worsen a depression status thereby decreasing sexual function and libido [21] (Fig. 3).

Since 1980s, several studies tried to assess a possible negative effect of different β-blockers on erectile function in men with essential hypertension, their results being highly controversial (Table 2, Ref. [22, 23, 24, 25, 26, 27]). Indeed, most trials did not treat the ED occurrence as a primary endpoint and sexual function was simply assessed by patient-reports instead of by means of a more objective evaluation such as measurement of penile rigidity or adoption of specific questionnaires [18]. In such a context, within a sort of survey on possible adverse events linked to antihypertensive drugs, propranolol were reported as more frequently related to sexual dysfunction than captopril or placebo [22, 23]. Similarly, in the late 1990s, Fogaro and colleagues demonstrated a worsening of sexual activity in newly diagnosed hypertensive patients treated with atenolol and carvedilol compared to lisinopril and valsartan, respectively [24, 25]. However, a systematic meta-analysis of randomized controlled trials including 35,000 patients found only a small increase in risk of sexual dysfunction with β-blocker therapy (5 per 1000 patients treated) [28]. Some authors tried to investigate the matter focusing on the different effects exerted by distinct β-blockers (i.e., pharmacologic profile and ancillary properties) (Table 3) and, accordingly, particularly for non-selective and lipophilic ones, such as propranolol, have been hypothesized a more evident effects on sexual behavior. Following the same reasoning, recent clinical trials reported beneficial effects of nebivolol, a third generation highly selective β_1_-blocker, devoid of intrinsic sympathomimetic properties, which, unlike classical β-blockers, has a vasodilator effect mediated by an increase in NO levels due to the activation of β_3_-adrenergic receptors. This effect might result in peripheral resistances reduction and, thereby, counteraction of endothelial dysfunction which may potentiate erectile response. In such a context, Doumas and colleagues reported that in hypertensive patients treated with atenolol, metoprolol or bisoprolol, switching treatment to nebivolol improved erectile function in 69% of cases after three months [29]. Strengthening the datum, the nebivolol did not lead to a sexual function worsening in hypertensive men with respect the atenolol and it showed a significantly greater beneficial effect on ED sub-scores and sexual activity than metoprolol, despite similar antihypertensive effectiveness [26, 30]. An observational trial evaluating more than 1000 hypertensive subjects treated with any β-blockers confirmed these results, concluding that ED is highly prevalent in patients treated with β-blockers, except for nebivolol-treated ones [27]. Nonetheless, it should be also cited an interesting study by Silvestri and colleagues which highlighted that the prejudice about this particular potential adverse effect of β-blockers therapy may itself result in ED through a mere psychological effect (i.e., Hawtorne effect) [31].

**Table 2.** **Main studies evaluating the association between β-blockers and erectile dysfunction (ED)**.

Reference	β-blocker tested	Type of study	Study sample	End points	Results
Peart WS *et al*. [22]	Propranolol	Randomized, single blind, placebo controlled	7513 men with mild to moderate essential hypertension	Death from hypertension or stroke and non-fatal stroke	Association between propranolol treatment and impotence
Croog SH *et al*. [23]	Propranolol	Randomized, double blind	626 men with mild to moderate essential hypertension	Effects on quality of life	Higher side effects and sexual dysfunction than captopril
Fogari R *et al*. [24]	Atenolol	Randomized, double blind	90 men with a newly diagnosed essential hypertension	Effects on sexual activity	Chronic worsening of sexual activity
Fogari R *et al*. [25]	Carvedilol	Randomized, double blind, placebo controlled	160 men with a newly diagnosed essential hypertension	Effects on sexual activity	Chronic worsening of sexual activity
Brixius K *et al*. [26]	Metoprolol/Nebivolol	Randomized, double blind	48 men with stage 1 essential hypertension	Effects on erectile function	Metoprolol decreased the erectile function/ Nebivolol improved it
Cordero A *et al*. [27]	Any β-blockade agent	Cross-sectional, observational	1007 men with essential hypertension	Prevalence of ED	ED is highly prevalent in hypertensive patients treated with β-blockers, except for Nebivolol

Note that all available studies deal with hypertensive patients.

**Table 3.** **Pharmacological differences in β-blockers’ agents**.

Name	Selectivity	Lipophilicity	Ancillary effects
Pindolol	Nonselective	Intermediate	Intrinsic sympathomimetic activity
Propranolol	Nonselective	High	Membrane stabilizing effect
Sotalol	Nonselective	Low	Type III antiarrhythmic action
Timolol	Nonselective	Intermediate	
Nadolol	Nonselective	Low	
Carvedilol*	Nonselective	Intermediate	α_1_-blocking and membrane stabilizing effect
Labetalol	Nonselective	High	α_1_-blocking activity
Penbutolol	Nonselective	High	Intrinsic sympathomimetic activity
Atenolol	β_1_-selective	Low	
Bisoprolol*	β_1_-selective	Intermediate	
Metoprolol*	β_1_-selective	Intermediate	
Nebivolol*	β_1_-selective	Low	β_3_ agonist activity
Esmolol	β_1_-selective	Low	
Celiprolol	β_1_-selective	Low	Intrinsic sympathomimetic activity
Acebutolol	β_1_-selective	Low	Intrinsic sympathomimetic activity

*β-blockers approved in heart failure therapy.

## 4. Other Heart Failure Therapies and Erectile Dysfunction

Most of the medications commonly used in HF therapy have been associated with sexual dysfunction but, again, data specifically obtained in such a population are lacking. Together with β-blockers, a significant role has been advocated for diuretics [18]. Spironolactone, the aldosterone antagonist currently used as standard HF therapy, may cause erectile failure as well as gynecomastia and a decrease in libido through its well-known antiandrogen effects [4]. Also, the thiazides chlorthalidone and hydrochlorothiazide were associated with worsening sexual function, the cause being unknown [32, 33]. Instead, the impact on ED of the renin angiotensin system-acting agents, another pivotal therapy in HFrEF, remains controversial, since ACE-i seems to have a neutral effect [23, 24, 34] and, even, several studies demonstrated that treatment with ARBs is associated with an improvement of sexual desire, frequency of sexual contacts and erectile function [25, 35, 36]. Indeed, angiotensin II is involved in detumescence of the corpus cavernosum and contribute to local oxidative stress. Therefore, by reducing its levels, ARBs might enhance endothelial function and promote vasorelaxation to improve erectile function. Finally, available data on the effect of digoxin on sexual function are very inconsistent but they suggest a possible association with ED, again the mechanism remaining not clearly understood [37].

## 5. Managing Erectile Dysfunction in HF

Since that management of the cardiac disease may reduce symptoms, improve exercise capacity and decrease depression, it is highly reasonable that targeting the HF therapy according to the current guidelines might be beneficial also with respect the sexual function. Notwithstanding, it would be highly desirable to investigate the presence of ED through specific questions since it can influence patients’ adherence to treatment or lead to misguided efforts to retain satisfactory sexual activity as well as adversely affect the quality of life [13]. In this setting, cardiologists should improve their engagement in managing patients with sexual function disorders [9]. They should inform their patients about the physiologic requirements of sexual activity as well as about the advantages of targeting HF therapy with respect to sexual function. Anyway, all drugs with potential adverse sexual side effects should be discontinued or replaced when clinically feasible. Furthermore, providing an adequate sexual counseling plays a pivotal role for the management of patients with HF and ED. Similarly, optimizing the treatment of cardiovascular risk factors, practicing regular exercise, losing weight, moderating alcohol consumption and quitting smoking are all essential steps. Indeed, lifestyle changes are essential to improve erectile function, to reduce the global cardiovascular risk burden and, most likely, to reduce most of the adverse drug effects [38, 39].

If the abovementioned measures are not still enough, a more specific approach to ED is recommended. In such a context, the PDE5 inhibitors, which block PDE-5 mediated degradation of cGMP thereby delaying detumescence, are the most commonly used drugs for treatment of ED. They have a modest hypotensive action and a mild nitrate-like action since PDE-5 is also present in vascular smooth muscle cells. Properly for their mechanisms, initially HF was considered as a relative contraindication for their use [40] but, subsequently, Katz and colleagues demonstrated the safety of this class of drugs in patients with mild to moderate HF [41]. In addition, several other potential hemodynamic benefits in HF patients were reported, such as a decrease in heart rate response during exercise, an improvement in exercise capacity and an increase in cardiac index [42, 43]. Thus, current guidelines consider the PDE-5 inhibitors generally safe in patients with compensated HF, except for those receiving nitrates [13] where it is possible an increased risk of symptomatic hypotension. Instead, there are no data supporting the efficacy of nutritional supplements, herbal therapy or vitamins in the treatment of ED. Yohimbine, an α_2_-receptor blocker with limited efficacy in the treatment of this condition, should be avoided in HF patients because of its cardiovascular side effects, including tachycardia and hypertension. Conversely, there are currently no known adverse effects to androgen replacement therapy, intra-urethral suppositories, penile prosthesis, or vacuum-assist erection devices when indicated [44].

## 6. Conclusions 

ED is a clinical condition highly prevalent among HF patients which adversely affect their quality of life. The tight association between these two conditions is most likely due to shared risk factors and common pathogenetic traits. HF itself may worsen sexual function for countless reasons, ranging from impaired exercise tolerance and psychogenic factors to neurohumoral, metabolic and vascular changes. Additionally, some cardiovascular medications might contribute to ED. Although evidence support a negative effect for diuretics and β-blockers, the available scientific literature is limited to hypertensive cohort and there are several methodological concerns with respect the ED evaluation. Conversely, it should be always kept in mind how a well-reasoned HF treatment according the current guidelines leads to undoubted advantages from a prognostic viewpoint as well as in terms of quality-of-life improvement. Nonetheless, particularly in the HF clinical setting, the ED-related issues require special care, being an effective treatment possible just pursuing the correction of reversible risk factors, the choice of more suitable medications and, in selected cases, by the use of specific drugs or devices.
